# Genetic variation in *Breviolum antillogorgium*, a coral reef symbiont, in response to temperature and nutrients

**DOI:** 10.1002/ece3.4959

**Published:** 2019-02-07

**Authors:** Shannon L. J. Bayliss, Zoë R. Scott, Mary Alice Coffroth, Casey P. terHorst

**Affiliations:** ^1^ Biology Department California State University Northridge California; ^2^ Department of Ecology and Evolutionary Biology University of Tennessee Knoxville Tennessee; ^3^ Department of Geology and Graduate Program in Evolution, Ecology and Behavior University at Buffalo Buffalo New York

**Keywords:** climate change, evolutionary rescue, genetic variation, mutualism, natural selection

## Abstract

Symbionts within the family *Symbiodiniaceae* are important on coral reefs because they provide significant amounts of carbon to many different reef species. The breakdown of this mutualism that occurs as a result of increasingly warmer ocean temperatures is a major threat to coral reef ecosystems globally. Recombination during sexual reproduction and high rates of somatic mutation can lead to increased genetic variation within symbiont species, which may provide the fuel for natural selection and adaptation. However, few studies have asked whether such variation in functional traits exists within these symbionts. We used several genotypes of two closely related species, *Breviolum antillogorgium* and *B. minutum*, to examine variation of traits related to symbiosis in response to increases in temperature or nitrogen availability in laboratory cultures. We found significant genetic variation within and among symbiont species in chlorophyll content, photosynthetic efficiency, and growth rate. Two genotypes showed decreases in traits in response to increased temperatures predicted by climate change, but one genotype responded positively. Similarly, some genotypes within a species responded positively to high‐nitrogen environments, such as those expected within hosts or eutrophication associated with global change, while other genotypes in the same species responded negatively, suggesting context‐dependency in the strength of mutualism. Such variation in traits implies that there is potential for natural selection on symbionts in response to temperature and nutrients, which could confer an adaptive advantage to the holobiont.

## 1 INTRODUCTION

Ecological interactions between symbiotic partners can vary from mutualistic to parasitic, depending on the environmental context of the interaction (Kiers, Palmer, Ives, Bruno, & Bronstein, 2010; Lesser, Stat, & Gates, 2013; Moran & Sloan, 2015; Sachs & Simms, 2006). In high‐resource environments, where hosts are not nutrient‐limited, symbionts may be more parasitic, despite providing large benefits to hosts when resources are limiting. The nature of the interaction between hosts and symbionts may also depend on the genetic identity or evolutionary history of one or both partners (Chong & Moran, 2016; Weese, Heath, Dentinger, & Lau, 2015). In a long‐term experiment that exposed legumes and rhizobia to high‐ and low‐nitrogen environments, rhizobial symbionts that evolved in high‐nitrogen environments shifted from mutualism toward parasitism in interactions with host clover species (*Trifolium* spp.) (Weese et al., 2015).

On coral reefs, mutualisms between unicellular dinoflagellates (Symbiodiniaceae) and reef species (e.g., corals and other cnidarians, mollusks, sponges, flatworms, foraminiferans) form the foundation of reefs. The algal cells provide photosynthetically derived sugars to the host in exchange for nitrogenous waste products (Lewis & Smith, 1971; Muscatine & Cernichiari, 1969; Muscatine & Porter, 1977; Trench, 1971a, 1971b, 1971c). When local temperatures exceed a threshold level, the symbiosis breaks down and “bleaching” occurs as a result of the loss of algal symbionts or pigmentation. Negative effects of increased temperatures associated with global change are already evident on reefs around the world, with bleaching events increasing in frequency (Donner, 2009; Eakin et al., 2010; Glynn, 1993; Hughes et al., 2017, 2018).

The likelihood of bleaching depends on host traits (Baird, Bhagooli, Ralph, & Takahashi, 2009 and references therein), but also on the traits of the symbiont (Quigley, Baker, Coffroth, Willis, & van Oppen, 2018 and references therein), and the conditions of the environment in which this interaction takes place (Anthony, Kline, Diaz‐Pulido, Dove, & Hoegh‐Guldberg, 2008; Harvell et al., 2007; Hoegh‐Guldberg, 1999). If symbiont traits can evolve in response to stressful environments, bleaching may be less likely to occur (Chakravarti, Beltran, & van Oppen, 2017; Chakravarti & van Oppen, 2018; Howells et al., 2012; van Oppen, Souter, Howells, Heyward, & Berkelmans, 2011; Zilber‐Rosenberg & Rosenberg, 2008). The short generation times of these microbial symbionts (1–2 days) offer the potential for evolution on ecological scales, given sufficient variation in the responses of different genotypes to environmental change.

Individual genetic variation in phenotypes provides the raw material for natural selection, which can lead to evolutionary rescue of populations from stressful conditions that would otherwise lead to local extinction (Gomulkiewicz & Holt, 1995). Mounting evidence suggests that genetic variation among individuals within a host species may affect bleaching response (Carilli, Donner, & Hartmann, 2012; Dixon et al., 2015; Kenkel et al., 2013; Pineda et al., 2013; Polato et al., 2010). Similarly, limited evidence suggests that, given sufficient genetic variation, symbionts can adapt to local conditions (Chakravarti et al., 2017; Chakravarti & van Oppen, 2018; Howells et al., 2012).

Given the rapid generation times of the symbionts, their evolutionary response may be more likely to lead to adaptation of the holobiont (hosts + symbionts). Quantitative PCR (qPCR) and microsatellites reveal numerous cases of multiple symbiont phylotypes and individual genotypes within a phylotype of symbiont in a single host (Correa, McDonald, & Baker, 2009; Howells, van Oppen, & Willis, 2009; Kirk, Andras, Harvell, Santos, & Coffroth, 2009; Mieog, Oppen, Berkelmans, Stam, & Olsen, 2009; Pettay & LaJeunesse, 2007; Santos, Gutierrez‐Rodriguez, & Coffroth, 2003; Silverman et al., 2012; Thornhill, Xiang, Fitt, & Santos, 2009). Theory suggests that bleaching may be adaptive if hosts switch to more temperature‐tolerant symbiont species or strains from the local environment, or shuffle symbionts such that more tolerant cryptic species become more abundant (Baker, 2003; Buddemeier & Fautin, 1993; Correa & Baker, 2011; Fautin & Buddemeier, 2004). However, host–symbiont pairings often revert to their original composition given sufficient time following a bleaching event, suggesting that not all strains are beneficial to hosts in all environments and/or that competitive advantage varies with environment (Coffroth, Poland, Petrou, Brazeau, & Holmberg, 2010; Jones, Berkelmans, van Oppen, Mieog, & Sinclair, 2008; LaJeunesse, Smith, Finney, & Oxenford, 2009; Lewis & Coffroth, 2004; Moran & Sloan, 2015; Thornhill, LaJeunesse, Kemp, Fitt, & Schmidt, 2006).

The family Symbiodiniaceae is taxonomically diverse and is comprised of over seven distinct genera (LaJeunesse et al., 2018) with substantial within‐genus variation representing groups of related species (Coffroth & Santos, 2005; LaJeunesse et al., 2018). Though many studies have examined functional trait diversity among and within Symbiodiniaceae genera (Frade, Bongaerts, Winkelhagen, Tonk, & Bak, 2008; Grégoire, Schmacka, Coffroth, & Karsten, 2017; Hennige, Suggett, Warner, McDougall, & Smith, 2009; Iglesias‐Prieto & Trench, 1994; Karim, Nakaema, & Hidaka, 2015; Krämer, Caamaño‐Ricken, Ricther, & Bischof, 2012; McGinty, Pieczoonka, & Mydlarz, 2012; Oakley, Schmidt, & Hopkinson, 2014; Ramsby, Shirur, Iglesias‐Prieto, & Goulet, 2014; Robison & Warner, 2006; Rodríguez‐Romän & Iglesias‐Prieto, 2005; Suggett et al., 2008; Takahashi, Whitney, & Badger, 2009), fewer studies have examined functional trait diversity among and within closely related species within the Symbiodiniaceae (Diaz‐Almeyda et al., 2017; Goyen et al., 2017; Klueter, Trapani, Archer, McIlroy, & Coffroth, 2017; Suggett et al., 2015). Functional trait variation is correlated with phylogenetic relatedness in some cases, but not in others, highlighting the need to examine functional trait variation at lower taxonomic scales (Suggett, Warner, & Leggat, 2017). In order for populations to evolve, such functional trait variation must exist within species (Chakravarti et al., 2017; Diaz‐Almeyda et al., 2017; Grégoire et al., 2017; Howells et al., 2012; Klueter et al., 2017; Parkinson & Baums, 2014). Such variation can arise during sexual reproduction and recombination, as well as by somatic mutations within a host (van Oppen et al., 2011). Mutation rates in rapidly asexually reproducing Symbiodiniaceae are high, relative to the time scale of coral growth; a symbiont population in a 30 cm coral colony is estimated to have acquired 780–78,000 beneficial mutations during development and growth (van Oppen et al., 2011).

Evidence suggests that symbiont populations can adapt to changes in temperature. Symbiont genotypes from warmer reefs performed better and promoted higher growth rates in hosts exposed to higher temperatures (Howells et al., 2012). Thermal tolerance traits can be highly heritable in symbiont populations, indicating that changes in symbiont performance following natural selection are likely to be passed on to the next generation after sexual reproduction (Császár, Ralph, Frankham, Berkelmans, & van Oppen, 2010; Quigley, Willis, & Bay, 2016). In fact, invasion of exapted symbiont genotypes into the Persian/Arabian Gulf resulted in strong selection that led to the dominance of *Cladocopium thermophilum* (formerly clade C, ITS2‐“Gulf C3”) genotypes throughout the Gulf (Hume et al., 2016). Similarly, stress‐tolerant *Durusdinium trenchii* has spread through the Caribbean Sea (Pettay, Wham, Smith, Iglesias‐Prieto, & LaJeaunesse, 2015). In a recent laboratory study, Chakravarti et al. (2017) found that *Cladocopium *C1 cultures subjected to laboratory selection at high temperature (31°C) had better photophysiology and growth at high temperature compared to wild‐type cells, suggesting that at least in some symbiont types, variation could allow a response to selection.

Here, we examine functional trait variation within the newly erected genus *Breviolum* (LaJeunesse et al., 2018) to examine whether genetic variation that could give rise to evolutionary rescue exists within species. Using several genotypes, we quantified functional traits that are most likely to affect the strength of interactions with the host. We ask whether the traits of different genotypes respond differently to increases in temperature or changes in the local nutrient environment to better understand the capacity of these populations to evolve in response to global change.

## 2 METHODS

### 2.1 Source of symbionts

We isolated two symbiont species within the genus *Breviolum *(B1‐ITS2 type) from the octocoral host, *Antillogorgium bipinnata,* from two locations within the Florida Keys (Looe Key and Tennessee Reef). We maintained the cultures in the Buffalo Undersea Reef Research Culture Collection for one to six years. Briefly, we collected ~3 cm from each of five host colonies; we preserved 1 cm in 95% ethanol for later molecular analysis of the dominant symbiont type within the host and ground the remaining 2 cm in 2 ml of filtered seawater (FSW). We poured the resultant slurry through a series of mesh filters (125, 74, and 20 µm) to remove larger pieces of host tissue and sclerites. We brought the homogenate to 10 ml with FSW and spun at 800 rpm for 5 min. The pellet was washed two more times with FSW and then resuspended in 1.0 ml of F/2 media (Guillard & Ryther, 1962). We added aliquots of 20 or 50 µl to each of six 50‐ml flasks with 30 ml of F/2 media. We maintained cultures under 40 W cool white lights with a 14:10‐hr light:dark cycle at 26°C and examined every 4–7 days for growth over a three‐month period. We transferred new *Breviolum *growth immediately to fresh media. Once growth was established, we transferred cultures to fresh media monthly and maintained cultures under the same conditions for three to nine years before imposing temperature and nutrient treatments and measuring traits.

In total, we identified seven distinct genotypes among our cultures (Table 1). Molecular analysis revealed that a subset of these were the symbiont *Breviolum antillogorgium, *the dominant symbiont within the host (Parkinson & Coffroth, 2015). Given that symbionts representative of the host are notoriously difficult to isolate in culture (LaJeunesse, 2002; Santos, Taylor, & Coffroth, 2001), the ability to isolate the dominant symbiont from this octocoral host makes *A. bipinnata* ideal for studying symbiont traits in culture that are also relevant for interactions with the host. A second symbiont species, *Breviolum minutum,* was represented by some cultures (Table 1), and we used these to examine both within‐ and between‐species variation in functional traits. All cultures came from different hosts, except 08‐0689.4 and 08‐0689.6, which came from the same host.

**Table 1:** Genotypes used in each experiment

Genotype^a^	Putative species	Expt. 1		Expt. 2		
		26°C	30°C	Low N	Medium N	High N
08.0689.4	*B. antillogorgium*	5	5			
08.0690.1	*B. minutum*	5	5			
08.0689.6	*B. minutum*	5	5	3	3	3
08‐0691.6	*B. minutum*			3	3	3
08‐0691.3	*B. antillogorgium*			3	3	3
13.117^b^	*B. antillogorgium*			3	3	2
13.143^b^	*B. antillogorgium*			3	3	3

Note

Numbers within cells represent the number of replicates that were uncontaminated and had sufficient cells to measure traits.

a See Supporting Information Table S1.

b Putatively the same genotype based on microsatellite loci and sequence analysis of B7 SYM15 flanking region and 23S rDNA (Table S1).

### 2.2 Molecular analysis

To determine symbiont species and genotype, symbiont DNA was extracted from each culture following the protocols of Coffroth, Lasker, Diamond, Bruenn, and Bermingham (1992). Species identification was based on sequence analysis of the flanking region of the B7Sym15 microsatellite and the chloroplast 23S rDNA (Parkinson & Coffroth, 2015) following the protocols of Thornhill, Xiang, Pettay, and Santos (2013) and Santos et al. (2002), respectively. PCR products were directly sequenced (TACGen, Richmond, CA), aligned in Geneious 6.1.8 using MUSCLE and phylogenetic tree generated in MAFFT using Jukes‐Cantor substitution mode and bootstrap values inferred from 1,000 replicates. Genotypes were determined based on five microsatellite loci (B7SYM15, B7SYM34, B7SYM36 following the protocols of Pettay & LaJeunesse, 2007; SYM155 following protocols of Andras, Kirk, Coffroth, & Harvell, 2009; and CA6.38 following the protocols of Santos, Shearer, Hannes, & Coffroth, 2004).

Before experiments were initiated, a subset of cultures representing different genotypes was transferred to California State University, Northridge, where stock cultures were maintained in 50–60 ml of F/2 culture medium in 125‐ml Erlenmeyer flasks. Cultures were maintained in a growth chamber with cool white lights (Philips 32W 700 series and Philips 17W T8 bulbs) on a 14:10‐hr light:dark cycle (daylight = 39 (±5.6) μmol photons m^−2^ s^−1^) at 26°C. Prior to experiments, we replaced 90% of the media once per month with sterile media. After the experiments described below, we isolated DNA from each culture and determined the genotypic composition of each culture to verify genotypes.

### 2.3 Experiment 1: Genotype responses to temperature

To test whether *Breviolum* genotypes differ in their response to temperature, we measured symbiont performance traits in culture at two temperatures. Five replicate cultures of each of three genotypes (Table 1) were maintained in growth chambers at either 26°C or 30°C. We inoculated replicate 50 ml cultures of F/2 media with appropriate genotypes at initial cell densities of 1 × 10^4^ cells/ml. This experiment consisted of 30 independent cultures (3 genotypes × 2 temperatures × 5 replicates; Table 1). We swirled flasks periodically to minimize settlement onto the glass surface. Every 7 days, we randomly rearranged cultures in the growth chamber to minimize any influence of spatial orientation within the growth chambers on the response. Preliminary evidence suggested that cultures peaked in abundance and reached steady‐state growth at 44 days (Supporting Information Figure S1), so after 39 days of growth, we used a well‐mixed 5 ml sample from each culture to quantify three performance traits (cell growth, photosynthetic efficiency, and chlorophyll concentration), as described below.

### 2.4 Experiment 2: Genotype responses to nitrogen

Because symbiont cells use the nitrogenous waste products of their host, the response of algal cells to nitrogen levels may alter their effectiveness as mutualist partners and the capacity for hosts to select symbionts. To test whether different genotypes of *Breviolum *differ in their response to nitrogen levels, we measured symbiont traits in culture at three nitrogen concentrations. Replicates of each of five genotypes were established in light and temperature conditions similar to the previous experiment, in growth chambers at 26°C. The F/2 medium in the 60 ml cultures was prepared at either normal (75 mg/L; 0.053 M; N:P ratio = 24.4), low (25 mg/L; 0.018 M; N:P ratio = 7.6), or high (150 mg/L; 0.098 M; N:P ratio = 48.7) nitrate concentrations. This experiment consisted of 45 independent cultures (5 genotypes × 3 nutrient environments × 3 replicates), though one replicate of genotype 13–117 at high nutrients failed to grow (Table 1). Cultures were initiated in 75‐cm^3^ Cell Culture Flasks (NEST^®^) at initial cell densities of 10^3^ cells/ml.

Cultures were maintained for 35 days. Each week we removed 10 ml of media and replaced it with media of the assigned nitrogen concentration, in order to maintain the nitrogen treatments. Although we did not quantify nitrogen levels in this experiment, changes in nitrate levels over one week (~1–5 mg/L) were considerably less than the differences among treatments (25, 75, and 150 mg/L). Before replacing media each week, we measured cell densities and used these to estimate population growth rate of cells in each culture. At the end of the experiment, we measured the same performance traits as in Experiment 1.

### 2.5 Performance measurements

At the end of each experiment, we mixed each culture well before removing 5 ml that we used to measure three performance traits: (a) cell growth, (b) photosynthetic efficiency, and (c) chlorophyll concentration. (a) We quantified cell density using an average of four counts of cell densities on a hemocytometer. Because each culture started at a known density, we used cell density after a given time as a rough proxy for population growth rate in Experiment 1. In Experiment 2, we used the average change in population size over four time periods as an estimate of growth rate. (b) To examine tolerance and acclimation of photosynthetic efficiency to environmental stress (i.e., elevated temperature and increase nutrients), we dark‐adapted a 2.5 ml sample of each replicate in a cuvette for 15 min before quantifying quantum yield (*Fv*/*Fm*) of photosystem II with an AquaPen‐C (Photon Systems Instruments). This measure provides an estimate of photosynthetic efficiency, where a decrease in dark‐adapted quantum yield (*Fv*/*Fm*) of photosystem II measured in the same organism in response to a treatment reflects a stress response to that treatment (Suggett et al., 2008). *Fv*/*Fm* may vary with cell size (Maxwell & Johnson, 2000; Suggett et al., 2015), but here we used two closely related species of similar cell size (*B. antillogorgium*: 7.1–8.1 µm, Parkinson & Coffroth, 2015 and *B. minutum*: 6.5–8.5 µm, LaJeunesse, Parkinson, & Reimer, 2012). (c) We used the same sample as above to measure in vivo chlorophyll *a* (Chl *a*) fluorescence on a Trilogy Laboratory Fluorometer (Turner Designs). As the Fluorometer has an upper limit in readable in vivo Chl *a*, 50% dilutions were used for samples that exceeded that limit by replacing 1.25 ml of sample with 1.25 ml of F/2 media. Chlorophyll concentrations were quantified as relative fluorescence units (RFU) and used to compare relative differences in Chl *a* concentrations between treatments, standardized by cell density.

### 2.6 Statistical analysis

We used trait data from each experiment in a principal component analysis (PCA) to visualize differences between genotypes in multidimensional space, using “princomp” in R version 3.3.2. For the PCA, we used performance traits measured at 26°C and normal F/2 nitrogen levels in each experiment, so that all traits were measured in the same environmental conditions. All traits were converted to z‐scores to meet assumptions of normality.

To examine differences in traits among genotypes and differential responses of genotypes to temperature, we used generalized linear models to examine treatment effects on each trait separately. Models included temperature, genotype, and their interactions as fixed factors. We used sample size‐corrected Akaike information criterion (AICc) and backwards stepwise selection to choose the best‐fit model for each variable. We examined differences among genotypes in response to nitrogen concentrations using similar analyses with nitrogen concentration as a fixed factor. We quantified the effects of each factor on: population growth rate, quantum yield, and chlorophyll concentration per cell.

## 3 RESULTS

### 3.1 Genetic variation in *Breviolum*

A subset of the cultures isolated from *Antillogorgia bipinnata* was assigned to two sister taxa (Clade B/ITS‐type B1), *Breviolum antillogorgium* and *B. minutum,* based on the approximately 530‐bp concatenated sequences of the flanking region of the B7Sym15 microsatellite and the chloroplast 23S rDNA (Supporting Information Figure S2). *B. antillogorgium* is a host‐specialist of *Antillogorgia* (Parkinson & Coffroth, 2015). *B. minutum* is the common symbiont in the anemone, *Exaiptasia*, and is most likely a transient species that can occur on the surface of or inside *Antillogorgia*, but is rarely the dominant symbiont type of this host. Microsatellite analysis yielded two to four alleles among the five microsatellite loci examined resulting in the identification of multiple distinct genotypes of *Breviolum *(Supporting Information Figure S2, Table S1).

**Figure 1:**
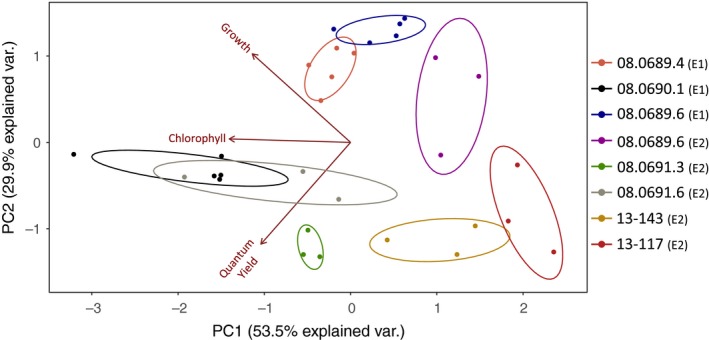
Biplot from principal components analysis of trait data of genotypes at ambient temperature and nitrogen levels in experiments 1 (E1) and 2 (E2). Each point represents a replicate culture, and different colors represent different genotypes

Most genotypes occupied unique spaces in multidimensional space (Figure 1). The first PC axis explained 54% of the variation in trait data and was primarily associated with chlorophyll per cell. The second PC axis explained 30% of the variation in trait data and was more associated with quantum yield and population growth rate.

### 3.2 Effects of temperature on traits

**Figure 2:**
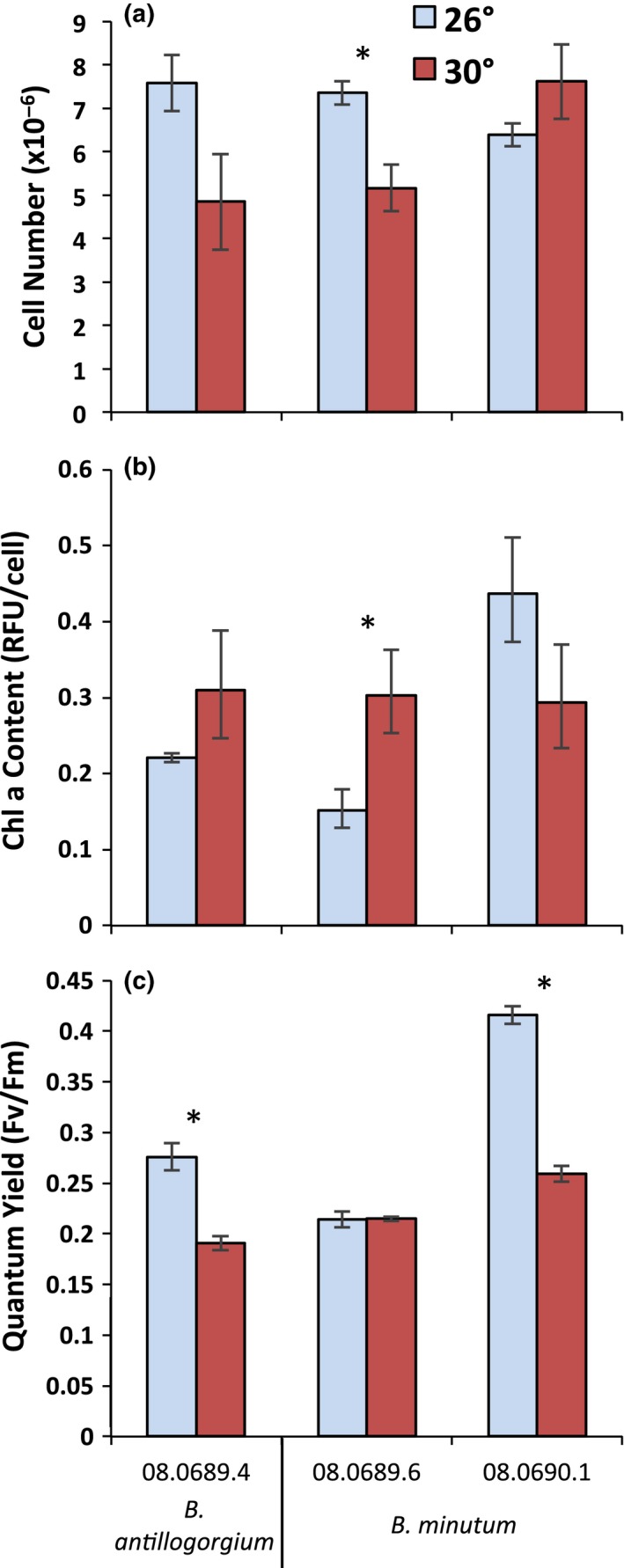
Mean (±*SE*) traits of one *Breviolum antillogorgium *genotype and two *Breviolum minutum* genotypes measured at two temperatures. Asterisks indicate genotypes with different trait values at different temperatures, based on Tukey pairwise comparisons

Temperature had a significant effect on the performance of *Breviolum *cultures, but the effects of temperature on traits were largely dependent on genotype. The response of cell density of different genotypes to increasing temperature varied significantly (Genotype*Temperature: *F*_2,24_ = 5.34, *p* = 0.012). Two genotypes, one *B. minutum* and one *B. antillogorgium*, responded negatively to increased temperature, but a different *B. minutum* genotype responded positively (Figure 2a).

The amount of chlorophyll per cell differed between temperatures, but again the response was dependent on genotype (Genotype*Temperature: *F*_2,24_ = 4.88, *p* = 0.017). One *B. minutum* genotype had less chlorophyll per cell at the higher temperature, but the other *B. minutum* genotype and the *B. antillogorgium* genotype had more chlorophyll per cell at the higher temperature (Figure 2b).

The response of quantum yield to increasing temperature also varied significantly among genotypes (Genotype*Temperature: *F*_2,24_ = 43.4, *p* < 0.0010). Genotypes with higher quantum yield at lower temperature showed a larger decrease in quantum yield at higher temperature (Figure 2c). The quantum yield of one *B. minutum* genotype decreased strongly at higher temperature. The *B. antillogorgium *genotype decreased less severely, and the other *B. minutum* genotype showed little change in quantum yield in response to temperature.

### 3.3 Effects of nutrients on traits

**Figure 3:**
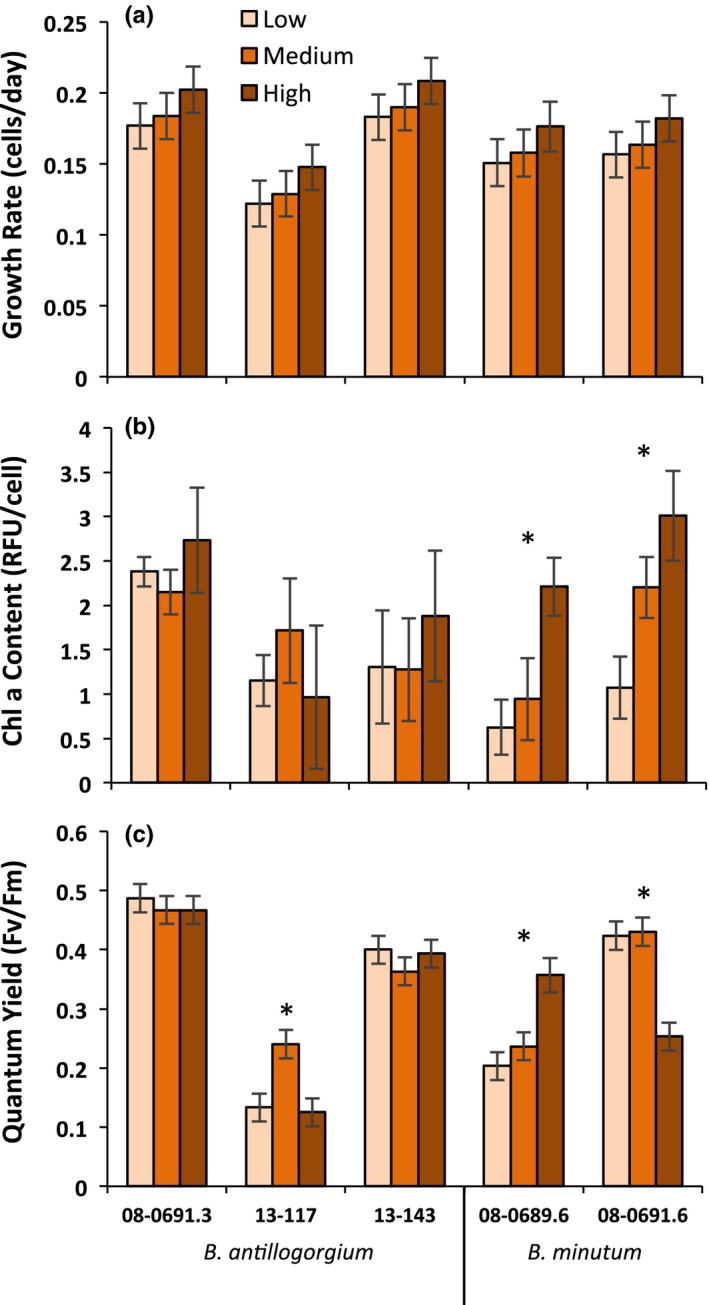
Mean (±*SE*) of three *Breviolum antillogorgium *genotypes and two *Breviolum minutum *genotypes measured at three different nitrogen concentrations: low (25 mg/L), medium (75 mg/L), and high (150 mg/L). Asterisks indicate genotypes with different trait values in different nitrogen environments, based on Tukey pairwise comparisons

The growth rates of genotypes were significantly different from one another (*F*_4,37_ = 2.92, *p* = 0.034), though there was no evidence that species were more different than genotypes (Figure 3a). Nitrogen had no significant effect on growth rates (*F*_2,37_ = 1.84, *p* = 0.174), and the Genotype*Nitrogen interaction was not a part of the best‐fit model (ΔAICc = 9.81).

The chlorophyll content of each genotype responded similarly to nitrogen treatments (*F*_8,29_ = 1.90, *p* = 0.099); however, chlorophyll content was significantly different among genotypes (*F*_4,37_ = 4.81, *p* = 0.004) and among nitrogen treatments (*F*_2,41_ = 6.31, *p* = 0.005). Tukey post hoc tests revealed that one *B. minutum* genotype (08‐0689.6) had significantly less chlorophyll than the other *B. minutum* genotype (08‐0691.6, *p* = 0.030) and one *B. antillogorgium* genotype (08‐0691.3, *p* < 0.001) (Figure 3b). Cells in the high‐nitrogen treatment had greater chlorophyll content than the low‐nitrogen treatment (*p* = 0.0019).

The response of quantum yield to nitrogen varied significantly among genotypes and within one putative genotype (cultures 13‐117 and 13‐143) (*F*_8,29_ = 8.83, *p* < 0.001). Two *B. antillogorgium* genotypes (08‐091.3 and 13‐143) showed little response of quantum yield to nitrogen. However, *B. antillogorgium* culture 13‐117, a putative clonemate of 13‐143 (Supporting Information Figure S2), had the highest quantum yield at the intermediate nitrogen level. One *B. minutum* genotype showed a decrease in quantum yield in response to increasing nitrogen, while the other *B. minutum* genotype showed an increase (Figure 3c).

## 4 DISCUSSION

Although taxonomists continue to partition the genetic variation in the family Symbiodiniaceae into genera and ultimately species (Coffroth & Santos, 2005; Hume et al., 2015; Jeong et al., 2014; LaJeunesse, Lee, Gil‐Agudelo, Knowlton, & Jeong, 2015; LaJeunesse et al., 2018, 2012; Parkinson & Coffroth, 2015), our study reveals that significant functional variation exists among and within two closely related species. Genotypes of *Breviolum antillogorgium* and *B. minutum* have significantly different responses to temperature and nitrogen. Traits that are likely to affect the strength of the symbiont's relationship with hosts, such as photosynthetic efficiency or growth rate, decreased at higher temperature or nitrogen concentrations in some genotypes, but were unaffected or increased in other genotypes. This suggests that the relationship between symbionts and hosts depends on the specific genetic composition of the symbiont population. Further, this variation implies that these symbiont traits have the potential to evolve in response to selective pressures of increased ocean temperatures associated with climate change, or in response to nitrogen concentrations within the host or in the water column, and that selection will be environment‐dependent.

The host (*Antillogorgia bipinnata*) from which the symbionts in this study were collected typically harbors one dominant symbiont, though others may be present at abundances too low to detect with our methodology, which can detect approximately 10–1,000 cells in a sample (Santos et al., 2003). However, it is noteworthy that other hosts can maintain a number of genotypes, species, and even genera within a host (Howells et al., 2009; Quigley et al., 2014; Rowan & Knowlton, 1995). Interestingly, two *Breviolum* genotypes (08‐0689.4 and 08‐0689.6) that were isolated from the same host, but were different species, had similar traits and similar responses to temperature in Experiment 1. Conversely, two cultures (13‐117 and 13‐143) that were identified as the same genotype based on our molecular data, showed significant variation in the traits that we measured, suggesting that they are likely different genotypes not detected by our microsatellites.

As clonal reproduction predominates within this symbiont family, most variation in *Breviolum *genotypes is likely the result of mutations, though genetic variation may be further increased or maintained by transposons, retrotransposons, tandem repeats, or recombination during sexual reproduction (Shoguchi et al., 2013). Because of the high mutation rate in Symbiodiniaceae (van Oppen et al., 2011), even hosts that initially harbor a single symbiont genotype may quickly accumulate genetic variation. Natural selection occurs when some genotypes outperform other genotypes under different environmental conditions. As many temperature tolerance traits are heritable (Császár et al., 2010; Quigley et al., 2016), natural selection is likely to lead to evolution of temperature tolerance in the symbiont population. Selection on the symbiont population could result in evolutionary rescue of the holobiont, allowing hosts to persist through periods of higher temperature (Baskett, Gaines, & Nisbet, 2009; Chakravarti & van Oppen, 2018; van Oppen, Oliver, Putnam, & Gates, 2015; van Oppen et al., 2011).

As with temperature, we found variation in the functional response of *Breviolum* genotypes to nitrogen concentrations, which may affect how populations of symbionts respond to eutrophication in the water column, or to different nitrogen environments within a host. In the case of both temperature and nitrogen, such functional variation could be indicative of existing niche partitioning allowing for genotypic coexistence, similar to niche partitioning among species (Chase & Leibold, 2003). Differences among genotypes could maintain genetic diversity in natural populations of *Breviolum *and other species within the Symbiodiniaceae, similar to results in other systems (salt marshes [Proffitt, Travis, & Edwards, 2003], sea grass communities [Reusch, Ehlers, Hammerli, & Worm, 2005], arboreal communities [Schweitzer et al., 2004], and plant–insect interactions [Johnson & Agrawal, 2005]).

An important consideration to whether the temperature‐ or nitrogen‐tolerant genotypes in this experiment would lead to holobiont adaptation is how well performance in laboratory cultures relates to performance in a host (Moran & Sloan, 2015). The traits we have measured are likely to be important for interactions with a host, but the exact effects are difficult to predict. For example, high growth rates may be beneficial to hosts as they allow the host to acquire large symbiont populations quickly, or recover from bleaching in a short time. However, if genotypes with high growth rates do not supply the host with adequate carbon, the relationship may be more parasitic and the symbiosis more likely to break down (Cunning & Baker, 2013). Previous work suggests symbiont physiology indeed differs in culture and in hosts (Bhagooli & Hidaka, 2003; Chakravarti et al., 2017; Howells et al., 2012; Ralph, Gademann, & Larkum, 2001). For symbiont evolution to result in holobiont adaptation, the symbionts must not only evolve in response to changing environmental conditions, but also continue a mutually beneficial relationship with the host. Although Chakravarti et al. (2017) found evidence for thermal adaptation in vitro, thermally selected strains of *Cladocopium *C1 had less of an effect when introduced into the host. Work to determine whether symbiont genotype responses to temperature in culture and in hosts differ quantitatively or qualitatively is ongoing.

Differing amounts and types of nitrogen available in vitro and in hospite may also affect the outcome of host–symbiont interactions. Nitrogen concentrations in F/2 media are quite high, and it is unlikely that nitrogen was limiting, even in our low‐nitrogen treatment. This could be one reason why we did not observe significant differences in growth rates among nitrogen treatments, on average. Experimental nutrient conditions ranged from 0.4–2.4 µM nitrate L^−1^, whereas waters surrounding most coral reefs are very low in dissolved inorganic nitrogen with measures of <1 µM/L (Fiore, Jarett, Olson, & Lesser, 2010; Tanaka, Miyajima, Koike, Hayashibara, & Ogawa, 2007). Corals may supplement nitrogen available to symbionts by as little as 0.264 µmol N cm^−1^ day^−1^, an amount that exceeds the growth needs of the algae (Falkowski, Dubinsky, Muscatine, & Mccloskey, 1993; Rees, 1991). Further, we manipulated nitrate, which many algae, including those in the Symbiodiniaceae, can use, but most of the nitrogenous waste produced by hosts is in the form of ammonium and is a preferred source of nitrogen (Grover, Maguer, Allemand, & Ferrier‐Pages, 2003). Some portion of the nitrate in cultures was likely reduced to other forms of nitrogen, although the extent to which this occurred was not quantified in this experiment. Bacteria also likely play in role in the abundance and forms of nitrogen available, and we do not yet know whether genotype traits differ because of specific genetic differences in the algae, or if different algal genotypes harbor different bacterial communities. Although future experiments should explore different quantities or forms of nitrogen, growing symbionts in culture at such low concentrations can be difficult, or at best, time consuming. Regardless, this work suggests that we are unlikely to understand the performance and response of hosts to eutrophication or other aspects of global change without accounting for genetic differences in the symbiont population.

The surprising amount of genetic and functional trait variation observed within and among these symbiont species, coupled with the short generation times of these organisms, suggests that populations of symbionts have the capacity to evolve over ecologically relevant time scales. Though hosts may evolve in response to global change, the rapid evolutionary potential of the symbionts with shorter generation times may be a faster route to adaptation for the holobiont. For example, Chakravarti and van Oppen (2018) found that symbiont populations grown at high temperatures began to outperform wild‐type symbionts in terms of growth rate and photosynthetic efficiency in as little 40–70 asexual generations. The potential for this group of symbionts to evolve offers some hope to the future of coral reefs. Evolutionary rescue may be an important mechanism by which species persist in the face of global change (Gomulkiewicz & Holt, 1995). Beyond evolution in the wild, others have called for assisted evolution by developing temperature‐tolerant strains of corals of critical conservation concern (Chakravarti & van Oppen, 2018; van Oppen et al., 2015). Though our research suggests that variation within species exists, allowing some scope for natural selection, the success of introducing adapted strains in natural populations will also require successful infection of hosts with those strains, growth inside the host, and the adapted symbionts must increase the fitness of the holobiont.

## CONFLICT OF INTEREST

None declared.

## AUTHOR CONTRIBUTIONS

SLJB, MAC, and CPt designed the experiments. SLJB and ZRS collected the data. SLJB and CPt analyzed the data. SLJB, ZRS, MAC, and CPt wrote the manuscript.

## Data Availability

Data from this manuscript are publicly available through the Biological & Chemical Oceanography Data Management Office (http://lod.bco-dmo.org/id/dataset/738212 and http://lod.bco-dmo.org/id/dataset/738228).

## References

[ece34959-bib-0001] Andras, J. P. , Kirk, N. L. , Coffroth, M. A. , & Harvell, C. D. (2009). Isolation and characterization of microsatellite loci in Symbiodinium B1/B184, the dinoflagellate symbiont of the Caribbean sea fan coral, *Gorgonia ventalina* . Molecular Ecology Resources, 9(3), 989–993. 10.1111/j.1755-0998.2009.02549.x 21564815

[ece34959-bib-0002] Anthony, K. R. N. , Kline, D. I. , Diaz‐Pulido, G. , Dove, S. , & Hoegh‐Guldberg, O. (2008). Ocean acidification causes bleaching and productivity loss in coral reef builders. Proceedings of the National Academy of Sciences of the United States of America, 105(45), 17442–17446. 10.1073/pnas.0804478105 18988740 PMC2580748

[ece34959-bib-0003] Baird, A. H. , Bhagooli, R. , Ralph, P. J. , & Takahashi, S. (2009). Coral bleaching: The role of the host. Trends in Ecology & Evolution, 24(1), 16–20. 10.1016/j.tree.2008.09.005 19022522

[ece34959-bib-0004] Baker, A. C. (2003). Flexibility and specificity in coral‐algal symbiosis: Diversity, ecology, and biogeography of Symbiodinium. Annual Review of Ecology Evolution and Systematics, 34, 661–689. 10.1146/annurev.ecolsys.34.011802.132417

[ece34959-bib-0005] Baskett, M. L. , Gaines, S. D. , & Nisbet, R. M. (2009). Symbiont diversity may help coral reefs survive moderate climate change. Ecological Applications, 19(1), 3–17. 10.1890/08-0139.1 19323170

[ece34959-bib-0006] Bhagooli, R. , & Hidaka, M. (2003). Comparison of stress susceptibility of in hospite and isolated zooxanthellae among five coral species. Journal of Experimental Marine Biology and Ecology, 291(2), 181–197. 10.1016/S0022-0981(03)00121-7

[ece34959-bib-0007] Buddemeier, R. , & Fautin, D. (1993). Coral bleaching as an adaptive mechanism: A testable hypothesis. BioScience, 43(5), 320–326. 10.2307/1312064

[ece34959-bib-0008] Carilli, J. , Donner, S. D. , & Hartmann, A. C. (2012). Historical temperature variability affects coral response to heat stress. PLoS ONE, 7(3), e34418 10.1371/journal.pone.0034418 22479626 PMC3316685

[ece34959-bib-0009] Chakravarti, L. J. , Beltran, V. H. , & van Oppen, M. J. H. (2017). Rapid thermal adaptation in photosymbionts of reef‐building corals. Global Change Biology, 23, 4675–4688. 28447372 10.1111/gcb.13702

[ece34959-bib-0010] Chakravarti, L. J. , & van Oppen, M. J. H. (2018). Experimental evolution in coral photosymbionts as a tool to increase thermal tolerance. Frontiers in Marine Science, 5, 227 10.3389/fmars.2018.00227

[ece34959-bib-0011] Chase, J. M. , & Leibold, M. A. (2003). Ecological niches: Linking classical and contemporary approaches. Chicago, IL: University of Chicago Press.

[ece34959-bib-0012] Chong, R. A. , & Moran, N. A. (2016). Intraspecific genetic variation in hosts affects regulation of obligate heritable symbionts. Proceedings of the National Academy of Sciences of the United States of America, 113(46), 13114–13119. 10.1073/pnas.1610749113 27799532 PMC5135297

[ece34959-bib-0013] Coffroth, M. , Lasker, H. , Diamond, M. , Bruenn, J. , & Bermingham, E. (1992). DNA fingerprints of a gorgonian coral: A method for detecting clonal structure in a vegetative species. Marine Biology, 114(2), 317–325. 10.1007/BF00349534

[ece34959-bib-0014] Coffroth, M. A. , Poland, D. M. , Petrou, E. L. , Brazeau, D. A. , & Holmberg, J. C. (2010). Environmental symbiont acquisition may not be the solution to warming seas for reef‐building corals. PLoS ONE, 5(10), e13258 10.1371/journal.pone.0013258 20949064 PMC2951366

[ece34959-bib-0015] Coffroth, M. A. , & Santos, S. R. (2005). Genetic diversity of symbiotic dinoflagellates in the genus *Symbiodinium* . Protist, 156(1), 19–34. 10.1016/j.protis.2005.02.004 16048130

[ece34959-bib-0016] Correa, A. M. S. , & Baker, A. C. (2011). Disaster taxa in microbially mediated metazoans: How endosymbionts and environmental catastrophes influence the adaptive capacity of reef corals. Global Change Biology, 17(1), 68–75. 10.1111/j.1365-2486.2010.02242.x

[ece34959-bib-0017] Correa, A. M. S. , McDonald, M. D. , & Baker, A. C. (2009). Development of clade‐specific *Symbiodinium* primers for quantitative PCR (qPCR) and their application to detecting clade D symbionts in Caribbean corals. Marine Biology, 156(11), 2403–2411. 10.1007/s00227-009-1263-5

[ece34959-bib-0018] Császár, N. B. M. , Ralph, P. J. , Frankham, R. , Berkelmans, R. , & van Oppen, M. J. H. (2010). Estimating the potential for adaptation of corals to climate warming. PLoS ONE, 5(3), e9751 10.1371/journal.pone.0009751 20305781 PMC2841186

[ece34959-bib-0019] Cunning, R. , & Baker, A. C. (2013). Excess algal symbionts increase the susceptibility of reef corals to bleaching. Nature Climate Change, 3(3), 259–262. 10.1038/nclimate1711

[ece34959-bib-0020] Diaz‐Almeyda, E. M. , Prada, C. , Ohdera, A. H. , Moran, H. , Civitello, D. J. , Iglesias‐Prieto, R. , … Medina, M. (2017). Intraspecific and interspecific variation in thermotolerance and photoacclimation in *Symbiodinium* dinoflagellates. Proceedings of the Royal Society B: Biological Sciences, 24(1868), 20171767. 10.1098/rspb.2017.1767 PMC5740277 29212723

[ece34959-bib-0021] Dixon, G. B. , Davies, S. W. , Aglyamova, G. A. , Meyer, E. , Bay, L. K. , & Matz, M. V. (2015). Genomic determinants of coral heat tolerance across latitudes. Science, 348(6242), 1460–1462. 10.1126/science.1261224 26113720

[ece34959-bib-0022] Donner, S. D. (2009). Coping with commitment: Projected thermal stress on coral reefs under different future scenarios. PLoS ONE, 4(6), e5712 10.1371/journal.pone.0005712 19492060 PMC2686172

[ece34959-bib-0023] Eakin, C. M. , Morgan, J. A. , Heron, S. F. , Smith, T. B. , Liu, G. , Alvarez‐Filip, L. , … Yusuf, Y. (2010). Caribbean corals in crisis: record thermal stress, bleaching, and mortality in 2005. PLoS ONE, 5, e13969 10.1371/journal.pone.0013969 21125021 PMC2981599

[ece34959-bib-0024] Falkowski, P. G. , Dubinsky, Z. , Muscatine, L. , & Mccloskey, L. (1993). Population control in symbiotic corals. BioScience, 43(9), 606–611. 10.2307/1312147

[ece34959-bib-0025] Fautin, D. G. , & Buddemeier, R. W. (2004). Adaptive bleaching: A general phenomenon. Hydrobiologia, 530, 459–467. 10.1007/s10750-004-2642-z

[ece34959-bib-0026] Fiore, C. L. , Jarett, J. K. , Olson, N. D. , & Lesser, M. P. (2010). Nitrogen fixation and nitrogen transformations in marine symbioses. Trends in Microbiology, 18(10), 455–463. 10.1016/j.tim.2010.07.001 20674366

[ece34959-bib-0027] Frade, P. R. , Bongaerts, P. , Winkelhagen, A. J. S. , Tonk, L. , & Bak, R. P. M. (2008). In situ photobiology of corals over large depth ranges: A multivariate analysis on the roles of environment, host and algal symbiont. Limnology and Oceanography, 53(6), 2711–2723. 10.4319/lo.2008.53.6.2711

[ece34959-bib-0028] Glynn, P. (1993). Coral reef bleaching: Ecological perspectives. Coral Reefs, 12(1), 1–17. 10.1007/BF00303779

[ece34959-bib-0029] Gomulkiewicz, R. , & Holt, R. (1995). When does evolution by natural selection prevent extinction. Evolution, 49(1), 201–207. 10.2307/2410305 28593677

[ece34959-bib-0030] Goyen, S. , Pernice, M. , Szabo, M. , Warner, M. E. , Ralph, P. J. , & Suggett, D. J. (2017). A molecular physiology basis for functional diversity of hydrogen peroxide production amongst *Symbiodinium* spp. (Dinophyceae). Marine Biology, 164(3), 46 10.1007/s00227-017-3073-5

[ece34959-bib-0031] Grégoire, V. , Schmacka, F. , Coffroth, M. A. , & Karsten, U. (2017). Photophysiological and thermal tolerance of various genotypes of the coral endosymbiont *Symbiodinium* sp. (Dinophyceae). Journal of Applied Phycology, 29(4), 1893–1905. 10.1007/s10811-017-1127-1

[ece34959-bib-0032] Grover, R. , Maguer, J. F. , Allemand, D. , & Ferrier‐Pages, C. (2003). Nitrate uptake in the scleractinian coral *Stylophora pistillata* . Limnology and Oceanography, 48(6), 2266–2274.

[ece34959-bib-0033] Guillard, R. , & Ryther, J. (1962). Studies of marine planktonic diatoms. 1. Cyclotella Nana Hustedt, and Detonula Confervacea (cleve) Gran. Canadian Journal of Microbiology, 8(2), 229–239. 13902807 10.1139/m62-029

[ece34959-bib-0034] Harvell, D. , Jordan‐Dahlgren, E. , Merkel, S. , Rosenberg, E. , Raymundo, L. , Smith, G. , … Willis, B. (2007). Coral disease, environmental drivers, and the balance between coral and microbial associates. Oceanography, 20(1), 172–195. 10.5670/oceanog.2007.91

[ece34959-bib-0035] Hennige, S. J. , Suggett, D. J. , Warner, M. E. , McDougall, K. E. , & Smith, D. J. (2009). Photobiology of *Symbiodinium* revisited: Bio‐physical and bio‐optical signatures. Coral Reefs, 28(1), 179–195. 10.1007/s00338-008-0444-x

[ece34959-bib-0036] Hoegh‐Guldberg, O. (1999). Climate change, coral bleaching and the future of the world's coral reefs. Marine and Freshwater Research, 50(8), 839–866. 10.1071/MF99078

[ece34959-bib-0037] Howells, E. J. , Beltran, V. H. , Larsen, N. W. , Bay, L. K. , Willis, B. L. , & van Oppen, M. J. H. (2012). Coral thermal tolerance shaped by local adaptation of photosymbionts. Nature Climate Change, 2(2), 116–120. 10.1038/NCLIMATE1330

[ece34959-bib-0038] Howells, E. J. , van Oppen, M. J. H. , & Willis, B. L. (2009). High genetic differentiation and cross‐shelf patterns of genetic diversity among Great Barrier Reef populations of *Symbiodinium* . Coral Reefs, 28(1), 215–225. 10.1007/s00338-008-0450-z

[ece34959-bib-0039] Hughes, T. P. , Kerry, J. T. , Álvarez‐Noriega, M. , Álvarez‐Romero, J. G. , Anderson, K. D. , Baird, A. H. , … Wilson, S. K. (2017). Global warming and recurrent mass bleaching of corals. Nature, 543, 373–377. 10.1038/nature21707 28300113

[ece34959-bib-0040] Hughes, T. P. , Kerry, J. T. , Baird, A. H. , Connolly, S. R. , Dietzel, A. , Eakin, C. M. , … Torda, G. (2018). Global warming transforms coral reef assemblages. Nature, 556, 492–496. 10.1038/s41586-018-0041-2 29670282

[ece34959-bib-0041] Hume, B. C. C. , D'Angelo, C. , Smith, E. G. , Stevens, J. R. , Burt, J. , & Wiedenmann, J. (2015). *Symbiodinium thermophilum* sp nov., a thermotolerant symbiotic alga prevalent in corals of the world’s hottest sea, the Persian/Arabian Gulf. Scientific Reports, 5, 8562. 25720577 10.1038/srep08562 PMC4342558

[ece34959-bib-0042] Hume, B. C. C. , Voolstra, C. R. , Arif, C. , D'Angelo, C. , Burt, J. A. , Eyal, G. , … Wiedenmann, J. (2016). Ancestral genetic diversity associated with the rapid spread of stress‐tolerant coral symbionts in response to Holocene climate change. Proceedings of the National Academy of Sciences of the United States of America, 113(16), 4416–4421. 10.1073/pnas.1601910113 27044109 PMC4843444

[ece34959-bib-0043] Iglesias‐Prieto, R. , & Trench, R. K. (1994). Acclimation and adaptation to irradiance in symbiotic dinoflagellates. I. Responses of the photosynthetic unit to changes in photon flus density. Marine Ecology Progress Series, 113, 163–175.

[ece34959-bib-0044] Jeong, H. J. , Jang, S. H. , Moestrup, O. , Kang, N. S. , Lee, S. Y. , Potvin, E. , & Noh, J. H. (2014). *Ansanella granifera* gen. et sp nov (Dinophyceae), a new dinoflagellate from the coastal waters of Korea. Algae, 29(2), 75–99. 10.4490/algae.2014.29.2.075

[ece34959-bib-0045] Johnson, M. T. J. , & Agrawal, A. A. (2005). Plant genotype and environment interact to shape a diverse arthropod community on evening primrose (*Oenothera biennis*). Ecology, 86(4), 874–885. 10.1890/04-1068

[ece34959-bib-0046] Jones, A. M. , Berkelmans, R. , van Oppen, M. J. H. , Mieog, J. C. , & Sinclair, W. (2008). A community change in the algal endosymbionts of a scleractinian coral following a natural bleaching event: Field evidence of acclimatization. Proceedings of the Royal Society Series B: Biological Sciences, 275(1641), 1359–1365. 10.1098/rspb.2008.0069 PMC2367621 18348962

[ece34959-bib-0047] Karim, W. , Nakaema, S. , & Hidaka, M. (2015). Temperature effects on the growth rates and photosynthetic activities of *Symbiodinium *cells. Journal of Marine Science and Engineering, 3, 368–381.

[ece34959-bib-0048] Kenkel, C. D. , Goodbody‐Gringley, G. , Caillaud, D. , Davies, S. W. , Bartels, E. , & Matz, M. V. (2013). Evidence for a host role in thermotolerance divergence between populations of the mustard hill coral (*Porites astreoides*) from different reef environments. Molecular Ecology, 22(16), 4335–4348. 10.1111/mec.12391 23906315

[ece34959-bib-0049] Kiers, E. T. , Palmer, T. M. , Ives, A. R. , Bruno, J. F. , & Bronstein, J. L. (2010). Mutualisms in a changing world: An evolutionary perspective. Ecology Letters, 13(12), 1459–1474. 10.1111/j.1461-0248.2010.01538.x 20955506

[ece34959-bib-0050] Kirk, N. L. , Andras, J. P. , Harvell, C. D. , Santos, S. R. , & Coffroth, M. A. (2009). Population structure of *Symbiodinium* sp associated with the common sea fan, *Gorgonia ventalina*, in the Florida Keys across distance, depth, and time. Marine Biology, 156(8), 1609–1623. 10.1007/s00227-009-1196-z

[ece34959-bib-0051] Klueter, A. , Trapani, J. , Archer, F. I. , McIlroy, S. E. , & Coffroth, M. A. (2017). Comparative growth rates of cultured marine dinoflagellates in the genus *Symbiodinium *and the effect of temperature and light. PLoS ONE, 12(11), e187707. 10.1371/journal.pone.0187707 PMC5706665 29186143

[ece34959-bib-0052] Krämer, W. E. , Caamaño‐Ricken, I. , Ricther, C. , & Bischof , K. (2012). Dynamic regulation of photoproteciton determines thermal tolerance of two phylotypes of two phylotypes of Symbiodiniu Clade A at two photon fluence rates. Photochemistry and Photobiology, 88, 398–413. 22117932 10.1111/j.1751-1097.2011.01048.x

[ece34959-bib-0053] LaJeunesse, T. C. (2002). Diversity and community structure of symbiotic dinoflagellates from Caribbean Reefs. Marine Biology, 141, 387–400.

[ece34959-bib-0054] LaJeunesse, T. C. , Lee, S. Y. , Gil‐Agudelo, D. L. , Knowlton, N. , & Jeong, H. J. (2015). *Symbiodinium necroappetens* sp nov (Dinophyceae): An opportunist “zooxanthella” found in bleached and diseased tissues of Caribbean reef corals. European Journal of Phycology, 50(2), 223–238. 10.1080/09670262.2015.1025857

[ece34959-bib-0055] LaJeunesse, T. C. , Parkinson, J. E. , Gabrielson, P. W. , Jeong, H. J. , Reimer, J. D. , Voolstra, C. R. , & Santos, S. R. (2018). Systematic revision of Symbiodiniaceae highlights the antiquity and diversity of coral endosymbionts. Current Biology, 28(16), 2570–2580.e6. 10.1016/j.cub.2018.07.00 30100341

[ece34959-bib-0056] LaJeunesse, T. C. , Parkinson, J. E. , & Reimer, J. D. (2012). A genetics‐based description of *Symbiodinium minutum* sp. nov. and *S. psygmophilum* sp. nov. (dinophyceae), two dinoflagellates symbiotic with cnidaria. Journal of Phycology, 48(6), 1380–1391. 10.1111/j.1529-8817.2012.01217.x 27008273

[ece34959-bib-0057] LaJeunesse, T. C. , Smith, R. T. , Finney, J. , & Oxenford, H. (2009). Outbreak and persistence of opportunistic symbiotic dinoflagellates during the 2005 Caribbean mass coral “bleaching” event. Proceedings of the Royal Society Series B: Biological Sciences, 276(1676), 4139–4148. 10.1098/rspb.2009.1405 PMC2821356 19740874

[ece34959-bib-0058] Lesser, M. P. , Stat, M. , & Gates, R. D. (2013). The endosymbiotic dinoflagellates (*Symbiodinium* sp.) of corals are parasites and mutualists. Coral Reefs, 32(3), 603–611. 10.1007/s00338-013-1051-z

[ece34959-bib-0059] Lewis, C. L. , & Coffroth, M. A. (2004). The acquisition of exogenous algal symbionts by an octocoral after bleaching. Science, 304(5676), 1490–1492. 10.1126/science.1097323 15178798

[ece34959-bib-0060] Lewis, D. , & Smith, D. (1971). Autotrophic nutrition of symbiotic marine coelenterates with special reference to hermatypic corals 1. Movement of photosynthetic products between symbionts. Proceedings of the Royal Society Series B: Biological Sciences, 178(1050), 111 10.1098/rspb.1971.0055

[ece34959-bib-0061] Maxwell, K. , & Johnson, G. N. (2000). Chlorophyll fluorescence: A practical guide. Journal of Experimental Botany, 51(345), 659–668. 10.1093/jexbot/51.345.659 10938857

[ece34959-bib-0062] McGinty, E. S. , Pieczoonka, J. , & Mydlarz, L. D. (2012). Variaitons in reactive oxygen release and antioxidant activity in multiple *Symbiodinium* types in response to elevated temperature. Microbial Ecology, 64, 1000–1007. 22767124 10.1007/s00248-012-0085-z

[ece34959-bib-0063] Mieog, J. C. , Van Oppen, M. J. H. , Berkelmans, R. , Stam, W. T. , & Olsen, J. L. (2009). Quantification of algal endosymbionts (*Symbiodinium*) in coral tissue using real‐time PCR. Molecular Ecology Resources, 9(1), 74–82. 10.1111/j.1755-0998.2008.02222.x 21564569

[ece34959-bib-0064] Moran, N. A. , & Sloan, D. B. (2015). The hologenome concept: Helpful or hollow? PLOS Biology, 13(12), e1002311. 26636661 10.1371/journal.pbio.1002311 PMC4670207

[ece34959-bib-0065] Muscatine, L. , & Cernichiari, E. (1969). Assimilation of photosynthetic products of zooxanthellae by a reef coral. Biological Bulletin, 137(3), 506–523. 10.2307/1540172 28368714

[ece34959-bib-0066] Muscatine, L. , & Porter, J. (1977). Reef corals: Mutualistic symbioses adapted to nutrient‐poor environments. BioScience, 27(7), 454–460. 10.2307/1297526

[ece34959-bib-0067] Oakley, C. A. , Schmidt, G. W. , & Hopkinson, B. M. (2014). Thermal responses of Symbiodinium photosynthetic carbon assimilation. Coral Reefs, 33, 501–512.

[ece34959-bib-0068] Parkinson, J. E. , & Baums, I. B. (2014). The extended phenotypes of marine symbioses: Ecological and evolutionary consequences of intraspecific genetic diversity in coral‐algal associations. Frontiers in Microbiology, 5, 445 10.3389/fmicb.2014.00445 25202306 PMC4142987

[ece34959-bib-0069] Parkinson, J. E. , & Coffroth, M. A. (2015). New species of clade B Symbiodinium (dinophyceae) from the greater Caribbean belong to different functional guilds: *S. aenigmaticum* Sp Nov., *S. antillogorgium* sp Nov., *S. endomadracis* sp Nov., and *S. pseudominutum* sp Nov. Journal of Phycology, 51(5), 850–858. 10.1111/jpy.12340 26986882

[ece34959-bib-0070] Pettay, D. T. , & Lajeunesse, T. C. (2007). Microsatellites from clade B *Symbiodinium* spp. specialized for Caribbean corals in the genus Madracis. Molecular Ecology Notes, 7(6), 1271–1274. 10.1111/j.1471-8286.2007.01852.x

[ece34959-bib-0071] Pettay, D. T. , Wham, D. C. , Smith, R. T. , Iglesias‐Prieto, R. , & LaJeaunesse, T. C. (2015). Microbial invasion of the Caribbean by an Indo‐Pacific coral zooxanthella. Proceedings of the National Academy of Sciences of the United States of America, 112(24), 7513–7518. 10.1073/pnas.1502283112 26034268 PMC4475936

[ece34959-bib-0072] Pineda, J. , Starczak, V. , Tarrant, A. , Blythe, J. , Davis, K. , Farrar, T. , … da Silva, J. C. B. (2013). Two spatial scales in a bleaching event: Corals from the mildest and the most extreme thermal environments escape mortality. Limnology and Oceanography, 58(5), 1531–1545. 10.4319/lo.2013.58.5.1531

[ece34959-bib-0073] Polato, N. R. , Voolstra, C. R. , Schnetzer, J. , DeSalvo, M. K. , Randall, C. J. , Szmant, A. M. , … Baums, I. B. (2010). Location‐specific responses to thermal stress in larvae of the reef‐building coral *Montastraea faveolata* . PLoS ONE, 5(6), e11221 10.1371/journal.pone.0011221 20585643 PMC2890407

[ece34959-bib-0074] Proffitt, C. E. , Travis, S. E. , & Edwards, K. R. (2003). Genotype and elevation influence *Spartina alterniflora* colonization and growth in a created salt marsh. Ecological Applications, 13(1), 180–192. 10.1890/1051-0761(2003)013[0180:GAEISA]2.0.CO;2

[ece34959-bib-0075] Quigley, K. M. , Baker, A. C. , Coffroth, M. A. , Willis, B. L. , & van Oppen, M. J. H. (2018). Bleaching resistance and the role of algal endosymbionts In Loudh J., & vanOppen M. (Eds.), Coral bleaching: Patterns, processes, causes and consequences (pp. 111–151). Springer Ecological Series. Berlin, Germany: Springer.

[ece34959-bib-0076] Quigley, K. M. , Davies, S. W. , Kenkel, C. D. , Willis, B. L. , Matz, M. V. , & Bay, L. K. (2014). Deep‐sequencing method for quantifying background abundances of *Symbiodinium* types: Exploring the rare *Symbiodinium* biosphere in reef‐building corals. PLoS ONE, 9(4), e94297 10.1371/journal.pone.0094297 24728373 PMC3984134

[ece34959-bib-0077] Quigley, K. M. , Willis, B. L. , & Bay, L. K. (2016). Maternal effects and *Symbiodinium* community composition drive differential patterns in juvenile survival in the coral *Acropora tenuis* . Royal Society Open Science, 3(10), 160471 10.1098/rsos.160471 27853562 PMC5098987

[ece34959-bib-0078] Ralph, P. J. , Gademann, R. , & Larkum, A. W. D. (2001). Zooxanthellae expelled from bleached corals at 33 degrees C are photosynthetically competent. Marine Ecology Progress Series, 220, 163–168. 10.3354/meps220163

[ece34959-bib-0079] Ramsby, B. D. , Shirur, K. P. , Iglesias‐Prieto, R. , & Goulet, T. L. (2014). *Symbiodinium *photosynthesis in Caribbean octocorals. PLoS ONE, 9(9), e106419 10.1371/journal.pone.0106419 25192405 PMC4156329

[ece34959-bib-0080] Rees, T. (1991). Are symbiotic algae nutrient deficient? Proceedings of the Royal Society B: Biological Sciences, 243(1308), 227–233. 10.1098/rspb.1991.0036

[ece34959-bib-0081] Reusch, T. B. H. , Ehlers, A. , Hammerli, A. , & Worm, B. (2005). Ecosystem recovery after climatic extremes enhanced by genotypic diversity. Proceedings of the National Academy of Sciences of the United States of America, 102(8), 2826–2831. 10.1073/pnas.0500008102 15710890 PMC549506

[ece34959-bib-0082] Robison, J. D. , & Warner, M. E. (2006). Differential impacts of photoacclimation and thermal stress on the photobiology of four different phylotypes of *Symbiodinium *(Pyrrhophyta). Journal of Phycology, 42, 568–579.

[ece34959-bib-0083] Rodríguez‐Romän, A. , & Iglesias‐Prieto, R. (2005). Regulation of photochemical activity in cultured symbiotic dinoflagellates under nitrate limitation and deprivation. Marine Biology, 146, 1063–1073.

[ece34959-bib-0084] Rowan, R. , & Knowlton, N. (1995). Intraspecific diversity and ecological zonation in coral algal symbiosis. Proceedings of the National Academy of Sciences of the United States of America, 92(7), 2850–2853. 10.1073/pnas.92.7.2850 7708736 PMC42316

[ece34959-bib-0085] Sachs, J. L. , & Simms, E. L. (2006). Pathways to mutualism breakdown. Trends in Ecology & Evolution, 21(10), 585–592. 10.1016/j.tree.2006.06.018 16828927

[ece34959-bib-0086] Santos, S. R. , Gutierrez‐Rodriguez, C. , & Coffroth, M. A. (2003). Phylogenetic identification of symbiotic dinoflagellates via length heteroplasmy in domain V of chloroplast large subunit (cp23S)‐ribosomal DNA sequences. Marine Biotechnology, 5(2), 130–140. 10.1007/s10126-002-0076-9 12876648

[ece34959-bib-0087] Santos, S. R. , Shearer, T. L. , Hannes, A. R. , & Coffroth, M. A. (2004). Fine‐scale diversity and specificity in the most prevalent lineage of symbiotic dinoflagellates (*Symbiodinium*, Dinophyceae) of the Caribbean. Molecular Ecology, 13(2), 459–469. 10.1046/j.1365-294X.2004.02058.x 14717900

[ece34959-bib-0088] Santos, S. R. , Taylor, D. J. , & Coffroth, M. A. (2001). Genetic comparisons of freshly isolated versus cultured symbiotic dinoflagellates: Implications for extrapolating to the intact symbiosis. Journal of Phycology, 37(5), 900–912. 10.1046/j.1529-8817.2001.00194.x

[ece34959-bib-0089] Santos, S. R. , Taylor, D. J. , Kinzie, R. A. III , Hidaka, M. , Sakai, K. , & Coffroth, M. A. (2002). Molecular phylogeny of symbiotic dinoflagellates inferred from partial chloroplast large subunit (23S)‐rDNA sequences. Molecular Phylogenetics and Evolution, 23, 97–111. 10.1016/S1055-7903(02)00010-6 12069543

[ece34959-bib-0090] Schweitzer, J. A. , Bailey, J. K. , Rehill, B. J. , Martinsen, G. D. , Hart, S. C. , Lindroth, R. L. , … Whitham, T. G. (2004). Genetically based trait in a dominant tree affects ecosystem processes. Ecology Letters, 7(2), 127–134. 10.1111/j.1461-0248.2003.00562.x

[ece34959-bib-0091] Shoguchi, E. , Shinzato, C. , Kawashima, T. , Gyoja, F. , Mungpakdee, S. , Koyanagi, R. , … Satoh, N. (2013). Draft assembly of the *Symbiodinium minutum* nuclear genome reveals dinoflagellate gene structure. Current Biology, 23(15), 1399–1408. 10.1016/j.cub.2013.05.062 23850284

[ece34959-bib-0092] Silverman, J. , Kline, D. I. , Johnson, L. , Rivlin, T. , Schneider, K. , Erez, J. , … Caldeira, K. (2012). Carbon turnover rates in the One Tree Island reef: A 40‐year perspective. Journal of Geophysical Research‐Biogeosciences, 117, G03023 10.1029/2012JG001974

[ece34959-bib-0093] Suggett, D. J. , Goyen, S. , Evenhuis, C. , Szabo, M. , Pettay, D. T. , Warner, M. E. , & Ralph, P. J. (2015). Functional diversity of photobiological traits within the genus *Symbiodinium* appears to be governed by the interaction of cell size with cladal designation. New Phytologist, 208(2), 370–381. 26017701 10.1111/nph.13483

[ece34959-bib-0094] Suggett, D. J. , Warner, M. E. , & Leggat, W. (2017). Symbiotic dinoflagellate functional diversity mediates coral survival under ecological crisis. Trends in Ecology & Evolution, 32(10), 735–745. 10.1016/j.tree.2017.07.013 28843439

[ece34959-bib-0095] Suggett, D. J. , Warner, M. E. , Smith, D. J. , Davey, P. , Hennige, S. , & Baker, N. R. (2008). Photosynthesis and production of hydrogen peroxide by *Symbiodinium* (Pyrrhophyta) phylotypes with different thermal tolerances. Journal of Phycology, 44(4), 948–956. 27041613 10.1111/j.1529-8817.2008.00537.x

[ece34959-bib-0096] Takahashi, S. , Whitney, S. M. , & Badger, M. R. (2009). Different thermal sensitivity of the repair of photodamaged photosynthetic machinery in cultured *Symbiodinium* species. Proceedings of the National Academy of Sciences of the United States of America, 106(9), 3237–3242. 10.1073/pnas.0808363106 19202067 PMC2651304

[ece34959-bib-0097] Tanaka, Y. , Miyajima, T. , Koike, I. , Hayashibara, T. , & Ogawa, H. (2007). Imbalanced coral growth between organic tissue and carbonate skeleton caused by nutrient enrichment. Limnology and Oceanography, 52(3), 1139–1146. 10.4319/lo.2007.52.3.1139

[ece34959-bib-0098] Thornhill, D. J. , LaJeunesse, T. C. , Kemp, D. W. , Fitt, W. K. , & Schmidt, G. W. (2006). Multi‐year, seasonal genotypic surveys of coral‐algal symbioses reveal prevalent stability or post‐bleaching reversion. Marine Biology, 148(4), 711–722. 10.1007/s00227-005-0114-2

[ece34959-bib-0099] Thornhill, D. J. , Xiang, Y. , Fitt, W. K. , & Santos, S. R. (2009). Reef endemism, host specificity and temporal stability in populations of symbiotic dinoflagellates from two ecologically dominant Caribbean corals. PLoS ONE, 4(7), e6262 10.1371/journal.pone.0006262 19603078 PMC2706050

[ece34959-bib-0100] Thornhill, D. J. , Xiang, Y. , Pettay, D. T. , & Santos, S. R. (2013). Population genetic data of a model symbiotic cnidarian system reveal remarkable symbiotic specificity and vectored introductions across ocean basins. Molecular Ecology, 22, 449904515 10.1111/mec.12416 23980764

[ece34959-bib-0101] Trench, R. (1971a). Physiology and biochemistry of zooxanthellae symbiotic with marine coelenterates. 1. Assimilation of photosynthetic products of zooxanthellae by two marine coelenterates. Proceedings of the Royal Society Series B: Biological Sciences, 177(1047), 225 10.1098/rspb.1971.0024

[ece34959-bib-0102] Trench, R. (1971b). Physiology and biochemistry of zooxanthellae symbiotic with marine coelenterates. 2. Liberation of fixed C‐14 by zooxanthellae in‐vitro. Proceedings of the Royal Society Series B‐Biological Sciences, 177(1047), 237 10.1098/rspb.1971.0025

[ece34959-bib-0103] Trench, R. (1971c). Physiology and biochemistry of zooxanthellae symbiotic with marine coelenterates. 3. Effect of homogenates of host tissues on excretion of photosynthetic products in‐vitro by zooxanthellae from two marine coelenterates. Proceedings of the Royal Society Series B‐Biological Sciences, 177(1047), 251 10.1098/rspb.1971.0026

[ece34959-bib-0104] van Oppen, M. J. H. , Oliver, J. K. , Putnam, H. M. , & Gates, R. D. (2015). Building coral reef resilience through assisted evolution. Proceedings of the National Academy of Sciences of the United States of America, 112(8), 2307–2313. 10.1073/pnas.1422301112 25646461 PMC4345611

[ece34959-bib-0105] van Oppen, M. J. H. , Souter, P. , Howells, E. J. , Heyward, A. , & Berkelmans, R. (2011). Novel genetic diversity through somatic mutations: Fuel for adaptation of reef corals? Diversity, 3, 405–423.

[ece34959-bib-0106] Weese, D. J. , Heath, K. D. , Dentinger, B. T. M. , & Lau, J. A. (2015). Long‐term nitrogen addition causes the evolution of less‐cooperative mutualists. Evolution, 69(3), 631–642. 10.1111/evo.12594 25565449

[ece34959-bib-0107] Zilber‐Rosenberg, I. , & Rosenberg, E. (2008). Role of microorganisms in the evolution of animals and plants: The hologenome theory of evolution. FEMS Microbiology Reviews, 32(5), 723–735. 10.1111/j.1574-6976.2008.00123.x 18549407

