# Supplementary material for: Genetic variation in Breviolum antillogorgium, a coral reef symbiont, in response to temperature and nutrients
Source: Ecol Evol. 2019 Feb 7;9(5):2803–13. doi: 10.1002/ece3.4959 (PMC6406013; doi:10.1002/ece3.4959)
Supplement: Supplementary file 1 [file ECE3-9-2803-s001.docx]

Table S1. Genotypes assigned based on alleles recovered at 5 loci for each culture used in the experiments. *B. ant.* = *Breviolum antillogogia*; *B. min.* = *Breviolum minutum*.

| - **Sample** | - **Putative Species** | - **Host** | - **Collection Location** | - **B7SYM 15** | - **B7Sym 34** | - **B7Sym 36** | - **SYM 155** | - **CA 6.38** |
| --- | --- | --- | --- | --- | --- | --- | --- | --- |
| - 08.0689.4 | - *B. ant* | - 08-0689 | - Looe Key, FL | - 261 | - 286 | - 183 | - 251 | - 118 |
| - 08-0691.3 | - *B. ant* | - 08-0691 | - Looe Key, FL | - 263 | - 286 | - 183 | - 263 | - 118 |
| - 13-117 | - *B. ant* | - bipin 3 | - Tennessee Reef, FL | - 263 | - 278 | - 193 | - 251 | - 118 |
| - 13-143 | - *B. ant* | - bipin 19 | - Tennessee Reef, FL | - 263 | - 278 | - 193 | - 251 | - 118 |
| - 08-0689.6 | - *B. min* | - 08-0689 | - Looe Key, FL | - 259 | - 286 | - 183 | - 251 | - 118 |
| - 08.0690.1 | - *B. min* | - 08-0690 | - Looe Key, FL | - 259 | - 286 | - 183 | - 260 | - 118 |
| - 08.0691.6 | - *B. min* | - 08-0691 | - Looe Key, FL | - 271 | - 278 | - 183 | - 274 | - 122 |

Table S2. Accession numbers for sequences of the flanking region of the B7Sym15 microsatellite and the chloroplast 23S rDNA used for samples in Figure S2.

| **Sample** | B7SYM15 flanking region | Chloroplast 23S rDNA |
| --- | --- | --- |
| 08.0689.4 | MK393738 | MK393745 |
| 08-0691.3 | MK393739 | MK393746 |
| 13-117 | MK393740 | MK393747 |
| 13-143 | MK393741 | MK393748 |
| 08-0689.6 | MK393742 | MK393749 |
| 08.0690.1 | MK393743 | MK393750 |
| 08.0691.6 | MK393744 | MK393751 |
| *B. antillogorgium* | KT149353.1 | AY035417.1 |
| *B. minutum* | JX263427.1 | AY055235 |
| *B. psygmophilium* | KT194047.1 | AY035420.1 |

Figure S1

Figure S1. Abundance of cells in replicate cultures (n=5) over 56 days. Abundance peaks at 44 days, but stable growth is reached at approximately 37 days.

Figure S2


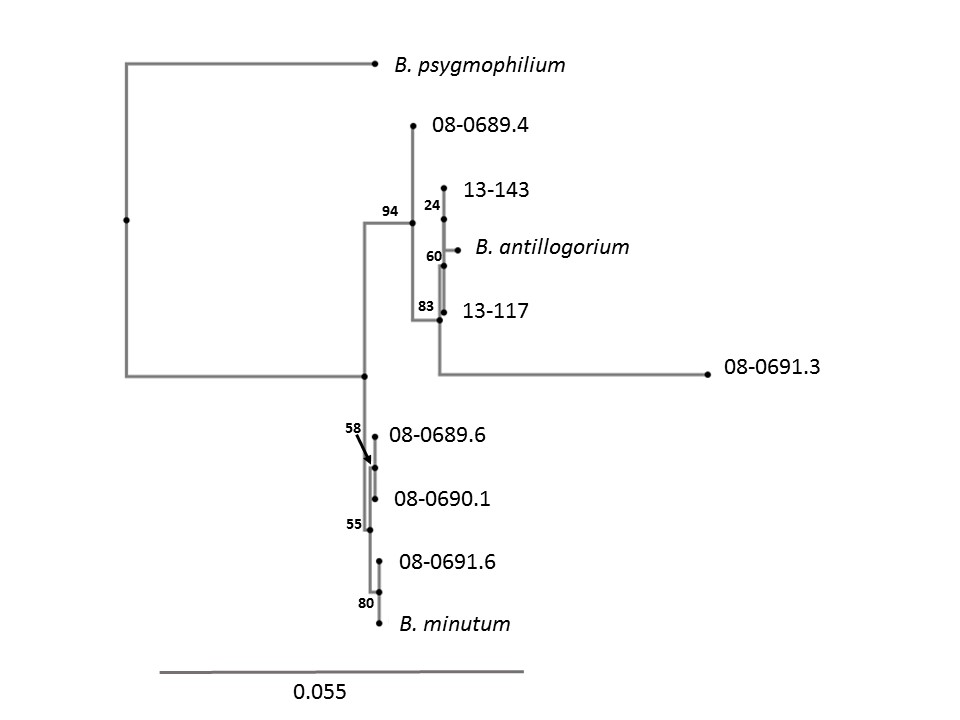


Figure S2. Molecular phylogenetic analysis by neighbor-joining in MAFFT using Jukes-Cantor substitution model with bootstrap resampling set at 1000. Bootstrap results shown next to branches. Branch length is proportional to distance (here based on sequence differences) with scale shown below the tree. Accession number for sequences given in Table S2.
